# A timeline of discovery and innovation in Arabidopsis

**DOI:** 10.1093/plcell/koaf108

**Published:** 2025-05-05

**Authors:** Catherine Freed, Arif Ashraf, Nancy A Eckardt, Adrienne H K Roeder, Joanna D Friesner

**Affiliations:** Department of Biochemistry, University of Wisconsin-Madison, Madison, WI 53706, USA; North American Arabidopsis Steering Committee, Corvallis, OR 97330, USA; North American Arabidopsis Steering Committee, Corvallis, OR 97330, USA; Department of Botany, University of British Columbia, Vancouver, BC, Canada V6T 1Z4; American Society of Plant Biologists, USA; North American Arabidopsis Steering Committee, Corvallis, OR 97330, USA; School of Integrative Plant Science, Section of Plant Biology and Weill Institute for Cell and Molecular Biology, Cornell University, Ithaca, NY 14853, USA; North American Arabidopsis Steering Committee, Corvallis, OR 97330, USA

To celebrate this Focus Issue on Translational Research from Arabidopsis to Crop Plants and Beyond, we present a timeline highlighting some of the exceptional discoveries, innovations, and community milestones in Arabidopsis research over the past 150+ years (Supplementary Figure S1). This timeline showcases multinational Arabidopsis efforts from as early as 1873 to the present, divided into five categories: technology, community, landmark paper, resource, and application. The technology category encompasses novel discoveries and research that have been particularly critical for advancing plant science into the 21st century. The community category showcases activities that helped to establish the Arabidopsis research community and enabled its evolution. The landmark paper category includes 25 of the top-cited Arabidopsis publications from *The Plant Cell* each year over the last 25 years. The resource category features new tools and resources developed by the Arabidopsis community, such as the creation of the Arabidopsis seed stock centers and online databases that have enabled plant researchers to gain a broader understanding of plant biology in Arabidopsis and other species. The application category highlights key technologies and plant products that have been made possible through fundamental Arabidopsis research efforts. This is not an exhaustive list, but a few examples that have made it to market. See the rest of the focus issue for other applications. Together, this timeline illustrates the rich history of Arabidopsis research and the Arabidopsis community, and the pivotal role Arabidopsis has played in advancing plant biology and developing the critical tools, resources, and technologies that continue to underpin innovations. Looking ahead, how will Arabidopsis-based research and discovery help to advance agriculture and biotechnology 25 years from now?

Landmark paper images are reproduced from *The Plant Cell* with permission.

1873—Technology—First Arabidopsis mutant published (Braun 1873; Yaschenko et al. 2024).

1935—Technology—N. Titova proposes Arabidopsis as a genetic tool (Titova 1935; Meyerowitz and Pruitt 1985).

1943- Technology—F. Laibach proposes Arabidopsis as a genetic tool (Laibach 1943; Meyerowitz and Pruitt 1985).

1947—Technology—X-Ray mutagenesis of Arabidopsis (Reinholz 1947).

1962—Technology—EMS mutagenesis of Arabidopsis seeds (Röbbelen 1962; McKelvie 1963).

1964—Community—The Arabidopsis Information Service—first community newsletter—founded.

1965—Resource—Early community Arabidopsis seed stock list (Röbbelen 1965).

1965—Community—First Arabidopsis community conference in Gottingen, Germany (Röbbelen 1965).

1975—Technology—G. Rédei proposes Arabidopsis as a genetic tool (Rédei 1975).

1982—Technology—First Arabidopsis plants in space (Merkys and Laurinavicius 1983).

1984—Technology—Arabidopsis has a small genome, good for positional cloning (Leutwiler et al. 1984).

1985—Community—Arabidopsis is recognized as a model system for basic research in plant molecular biology (Meyerowitz and Pruitt 1985).

1986—Technology—T-DNA-mediated transformation of Arabidopsis (An et al. 1986; Lloyd et al. 1986).

1987—Community—First modern era International Conference on Arabidopsis Research (ICAR) at Michigan State University, USA.

1987—Resource—Modern era community Arabidopsis seed stock list (Kranz and Kirchheim 1987).

1989—Technology—T-DNA mutagenesis (Feldmann et al. 1989; Marks and Feldmann 1989)

1990—Community—Multinational Arabidopsis Steering Committee (MASC) founded.

1990—Community—Broad Arabidopsis community email listserv (Bionet) founded.

1991—Resource—Major Arabidopsis Seed Stock centers (ABRC, USA & NASC, UK) established.

1992—Community—North American Arabidopsis Steering Committee (NAASC) founded.

1992—Resource—Arabidopsis database created (AAtDB) (Michael Cherry et al. 1992).

1994—Technology—Cloning of the first NLR innate immune receptors (Bent et al. 1994; Mindrinos et al. 1994  Grant et al. 1995).

1995—Technology—Microarrays to quantify whole Arabidopsis transcriptome (Schena et al. 1995).

1997—Technology—dsRNAi for gene silencing in Arabidopsis (Chuang and Meyerowitz 2000).

1997—Application—Paradigm Genetics and Mendel Biotechnology were both founded on the use of Arabidopsis genetics and genomics to derive function for translational use.

1998—Community—Arabidopsis is declared a model organism (Meinke et al. 1998).

1998—Technology—Arabidopsis floral dip with Agrobacterium tumefaciens (Clough and Bent 1998).

2000—Resource—Arabidopsis genome sequence is released. First plant/third eukaryote genome sequenced (The Arabidopsis Genome Initiative 2000).

2000—Community—Workshop Report: “The 2010 Project” Functional Genomics & the Virtual Plant. A Blueprint for Understanding How Plants Are Built & How to Improve Them (Chory et al. 2000).

2000—Landmark Paper—Hd1, a Major Photoperiod Sensitivity Quantitative Trait Locus in Rice, Is Closely Related to the Arabidopsis Flowering Time Gene CONSTANS (Yano et al. 2000).

2000—Technology—TILLING developed in Arabidopsis (McCallum et al. 2000).

2001—Landmark Paper—Growth Stage–Based Phenotypic Analysis of Arabidopsis: A Model for High Throughput Functional Genomics in Plants (Boyes et al. 2001).

2001—Resource—The Arabidopsis Information Resource (TAIR) released (Huala et al. 2001).

2002—Landmark Paper—Arabidopsis Transcriptome Profiling Indicates That Multiple Regulatory Pathways Are Activated during Cold Acclimation in Addition to the CBF Cold Response Pathway (Fowler and Thomashow 2002).

2003—Resource—Large-scale Arabidopsis T-DNA insertion line collection with locations sequenced (Alonso et al. 2003).

2003—Landmark Paper—Arabidopsis AtMYC2 (bHLH) and AtMYB2 (MYB) Function as Transcriptional Activators in Abscisic Acid Signaling (Abe et al. 2003).

2004—Landmark Paper—Novel and Stress-Regulated MicroRNAs and Other Small RNAs from Arabidopsis (Sunkar and Zhu 2004).

2005—Technology—Translating Ribosome Affinity Purification (TRAP) was first developed in Arabidopsis and then applied to other organisms (Zanetti et al. 2005).

2005—Landmark Paper—Functional Genomic Analysis of the AUXIN RESPONSE FACTOR Gene Family Members in *Arabidopsis thaliana*: Unique and Overlapping Functions of ARF7 and ARF19 (Okushima et al. 2005).

2006—Landmark Paper—First gene silencing by artificial miRNAs in Arabidopsis. Highly Specific Gene Silencing by Artificial MicroRNAs in Arabidopsis (Schwab et al. 2006).

2007—Technology—Arabidopsis Electronic Fluorescent Pictograph (eFP) Browser released (Winter et al. 2007).

2007—Landmark Paper—MYC2 Differentially Modulates Diverse Jasmonate-Dependent Functions in Arabidopsis (Dombrecht et al. 2007).

2008—Resource—The 1001 Genomes Project was launched to discover detailed whole-genome sequence variation in at least 1001 strains (accessions) of *Arabidopsis thaliana* (Alonso-Blanco et al. 2016).

2008—Landmark Paper—A Battery of Transcription Factors Involved in the Regulation of Secondary Cell Wall Biosynthesis in Arabidopsis (Zhong et al. 2008).

2009—Application—Auxin inducible degron (AID) system based on Arabidopsis auxin signaling, adapted to inducibly degrade target proteins in yeast & mammalian cells (Nishimura et al. 2009).

2009—Landmark Paper—MYB58 and MYB63 Are Transcriptional Activators of the Lignin Biosynthetic Pathway during Secondary Cell Wall Formation in Arabidopsis (Zhou et al. 2009).

2009—Application—Optogenetics: Light switchable protein interaction based on PHY-PIF system (Levskaya et al. 2009).

2010—Landmark Paper—The bHLH Transcription Factor POPEYE Regulates Response to Iron Deficiency in Arabidopsis Roots (Long et al. 2010).

2011—Technology—INTACT (isolation of nuclei tagged in specific cell types) developed in Arabidopsis (Deal and Henikoff 2011).

2011—Landmark Paper—The Arabidopsis bHLH Transcription Factors MYC3 and MYC4 Are Targets of JAZ Repressors and Act Additively with MYC2 in the Activation of Jasmonate Responses (Fernández-Calvo et al. 2011).

2012—Landmark Paper—Genome-Wide Analysis Uncovers Regulation of Long Intergenic Noncoding RNAs in Arabidopsis (Liu et al. 2012).

2013—Technology—Arabidopsis and Nicotiana were the first plants to be edited using CRISPR (Li et al. 2013).

2013—Landmark Paper—Jasmonate Regulates the INDUCER OF CBF EXPRESSION– C-REPEAT BINDING FACTOR/DRE BINDING FACTOR1 Cascade and Freezing Tolerance in Arabidopsis (Hu et al. 2013).

2014—Landmark Paper—Arabidopsis miR156 Regulates Tolerance to Recurring Environmental Stress through SPL Transcription Factors (Stief et al. 2014).

2015—Application—BASF introduced the PodGuard trait to Canola farmers which was developed by Bayer based on discoveries of the Arabidopsis INDEHISCENT gene (Liljegren et al. 2004; Liljegren and Yanofsky 2006; Aguilera 2019).

2015—Landmark Paper—A Cascade of Sequentially Expressed Sucrose Transporters in the Seed Coat and Endosperm Provides Nutrition for the Arabidopsis Embryo (Chen et al. 2015).

2015—Technology—Development of the MorphoGraphX image analysis software (Barbier de Reuille et al. 2015).

2016—Landmark Paper—Regulation of Leaf Starch Degradation by Abscisic Acid Is Important for Osmotic Stress Tolerance in Plants (Thalmann et al. 2016).

2016—Technology—DAP-seq developed in Arabidopsis (O’Malley et al. 2016).

2017—Landmark Paper—Arabidopsis WRKY46, WRKY54, and WRKY70 Transcription Factors Are Involved in Brassinosteroid-Regulated Plant Growth and Drought Responses (Chen et al. 2017).

2018—Landmark Paper—The m6A Reader ECT2 Controls Trichome Morphology by Affecting mRNA Stability in Arabidopsis (Wei et al. 2018).

2019—Application—First commercial production of CoverCress: field pennycress converted into a winter-annual oilseed crop based on Arabidopsis information (Phippen et al. 2022; Chopra et al. 2018, 2020).

2019—Landmark Paper—Dynamics of Gene Expression in Single Root Cells of *Arabidopsis thaliana* (Jean-Baptiste et al. 2019).

2019—Technology—Large-scale single cell transcriptomics in plants in Arabidopsis roots (Denyer et al. 2019; Jean-Baptiste et al. 2019; Ryu et al. 2019; Shulse et al. 2019; Zhang et al. 2019).

2020—Landmark Paper—Persulfidation-based Modification of Cysteine Desulfhydrase and the NADPH Oxidase RBOHD Controls Guard Cell Abscisic Acid Signaling (Shen et al. 2020).

2021—Landmark Paper—Distinct identities of leaf phloem cells revealed by single cell transcriptomics (Kim et al. 2021).

2021—Application—During 2000–2018 Corteva field-testing and screening for traits to improve crop tolerance to abiotic stress, over 90% of the 35,000 genes in the prescreening were identified in Arabidopsis. (Simmons et al. 2021).

2022—Landmark Paper—HPCA1 is required for systemic reactive oxygen species and calcium cell-to-cell signaling and plant acclimation to stress (Fichman et al. 2022).

2023—Application—Pairwise developed Conscious Greens, a nutritious and better tasting mustard green, using CRISPR to knock out the myrosinase genes to make them less bitter (Grinstein 2023). This was based on decades of research on glucosinolates and the myrosinase enzymes that break them down in Arabidopsis and other Brassicaceae plants (Halkier and Gershenzon 2006).

2023—Technology—Spatial single cell sequencing in Arabidopsis (Nobori et al. 2023).

2023—Landmark Paper—Auxin contributes to jasmonate-mediated regulation of abscisic acid signaling during seed germination in Arabidopsis (Mei et al. 2023).

2023—Application—Introduction of AtNPR1 into sweet orange trees to increase resistance to Huanglongbing (HLB) citrus greening in 2015 (Dutt et al. 2015). As of 2023, this technology is currently under regulatory status review by USDA APHIS (Silva et al. 2018; USDA APHIS 2023).

2024—Technology—Arabidopsis single cell proteomics (Montes et al. 2024).

2024—Landmark Paper—The m^6^A reader ECT1 drives mRNA sequestration to dampen salicylic acid–dependent stress responses in Arabidopsis (Lee et al. 2024).

2024—Application—Norfolk HP developed The Purple Tomato, bioengineered to have increased antioxidants. Arabidopsis-based gene discovery aided in development of purple tomato as the project started with the extraction of AtMYB12 protein from Arabidopsis and exploration revealed that AtMYB12 increases flavonoid accumulation and phenylpropanoid biosynthesis in tomato (Zhang et al. 2015). The Purple Tomato was released by Norfolk Healthy Produce as an FDA-approved product in 2023 and became available for purchase in 2024 (Martin and Butelli 2025).

2024—Community—First Arabidopsis community awards given (North American Arabidopsis Steering Committee).

## Supplementary Material

koaf108_Supplementary_Data

## Data Availability

No new data were generated or analyzed in support of this article.

## Supplementary data

The following materials are available in the online version of this article.

**Supplementary Figure S1.** Full size timeline pdf.

## Dive Curated Terms

The following phenotypic, genotypic, and functional terms are of significance to the work described in this paper:

ARF19 Gramene: AT1G19220
ARF19 Araport: AT1G19220

MYB63 Gramene: AT1G79180
MYB63 Araport: AT1G79180

SPL Gramene: AT4G27330
SPL Araport: AT4G27330

WRKY46 Gramene: At2g46400
WRKY46 Araport: At2g46400

WRKY54 Gramene: AT2G40750
WRKY54 Araport: AT2G40750

WRKY70 Gramene: AT3G56400
WRKY70 Araport: AT3G56400

ARF7 Gramene: AT5G20730
ARF7 Araport: AT5G20730

MYB2 Gramene: AT2G47190
MYB2 Araport: AT2G47190

MYC2 Gramene: AT1G32640
MYC2 Araport: AT1G32640

MYB58 Gramene: AT1G16490
MYB58 Araport: AT1G16490

POPEYE Gramene: AT3G47640
POPEYE Araport: AT3G47640

MYC3 Gramene: AT5G46760
MYC3 Araport: AT5G46760

MYC4 Gramene: AT4G17880
MYC4 Araport: AT4G17880

miR156A Gramene: AT2G25095
miR156A Araport: AT2G25095

INDEHISCENT Gramene: AT4G00120
INDEHISCENT Araport: AT4G00120

ECT2 Gramene: AT3G13460
ECT2 Araport: AT3G13460

RBOHD Gramene: AT5G47910
RBOHD Araport: AT5G47910

HPCA1 Gramene: AT5G49760
HPCA1 Araport: AT5G49760

ECT1 Gramene: AT3G03950
ECT1 Araport: AT3G03950

MYB12 Gramene: AT2G47460
MYB12 Araport: AT2G47460

## References

[koaf108-B1] Abe H, Urao T, Ito T, Seki M, Shinozaki K, Yamaguchi-Shinozaki K. Arabidopsis AtMYC2 (bHLH) and AtMYB2 (MYB) function as transcriptional activators in abscisic acid signaling. Plant Cell. 2003:15(1):63–78. 10.1105/tpc.006130 12509522 PMC143451

[koaf108-B2] Aguilera, MC. Shatterproof: the seeds of a blockbuster discovery. UC San Diego Today. 2019. https://today.ucsd.edu/story/shatterproof_the_seeds_of_a_blockbuster_discovery

[koaf108-B3] Alonso JM, Stepanova AN, Leisse TJ, Kim CJ, Chen H, Shinn P, Stevenson DK, Zimmerman J, Barajas P, Cheuk R, et al Genome-wide insertional mutagenesis of Arabidopsis thaliana. Science. 2003:301(5633):653–657. 10.1126/science.1086391 12893945

[koaf108-B4] Alonso-Blanco C, Andrade J, Becker C, Bemm F, Bergelson J, Borgwardt KM, Cao J, Chae E, Dezwaan TM, Ding W, et al 1,135 genomes reveal the global pattern of polymorphism in Arabidopsis thaliana. Cell. 2016:166(2):481–491. 10.1016/j.cell.2016.05.063 27293186 PMC4949382

[koaf108-B5] An G, Watson BD, Chiang CC. Transformation of tobacco, tomato, potato, and Arabidopsis thaliana using a binary ti vector system 1. Plant Physiol. 1986:81(1):301–305. 10.1104/pp.81.1.301 16664795 PMC1075324

[koaf108-B6] Barbier de Reuille P, Routier-Kierzkowska A-L, Kierzkowski D, Bassel GW, Schüpbach T, Tauriello G, Bajpai N, Strauss S, Weber A, Kiss A, et al MorphoGraphX: a platform for quantifying morphogenesis in 4D. Elife. 2015:4:e05864. 10.7554/eLife.05864 25946108 PMC4421794

[koaf108-B7] Bent AF, Kunkel BN, Dahlbeck D, Brown KL, Schmidt R, Giraudat J, Leung J, Staskawicz BJ. RPS2 of Arabidopsis thaliana: a leucine-rich repeat class of plant disease resistance genes. Science. 1994:265(5180):1856–1860. 10.1126/science.8091210 8091210

[koaf108-B8] Boyes DC, Zayed AM, Ascenzi R, McCaskill AJ, Hoffman NE, Davis KR, Görlach J. Growth stage–based phenotypic analysis of Arabidopsis: a model for high throughput functional genomics in plants. Plant Cell. 2001:13(7):1499–1510. 10.1105/tpc.010011 11449047 PMC139543

[koaf108-B9] Braun A . Sitzungsberichte der gesellschaft naturforschender freunde zu Berlin. Berlin: Gesellschaft Naturforschender Freunde; 1873.

[koaf108-B10] Chen J, Nolan TM, Ye H, Zhang M, Tong H, Xin P, Chu J, Chu C, Li Z, Yin Y. Arabidopsis WRKY46, WRKY54, and WRKY70 transcription factors are involved in brassinosteroid-regulated plant growth and drought responses. Plant Cell. 2017:29(6):1425–1439. 10.1105/tpc.17.00364 28576847 PMC5502465

[koaf108-B11] Chen L-Q, Lin IW, Qu X-Q, Sosso D, McFarlane HE, Londoño A, Samuels AL, Frommer WB. A cascade of sequentially expressed sucrose transporters in the seed coat and endosperm provides nutrition for the Arabidopsis embryo. Plant Cell. 2015:27(3):607–619. 10.1105/tpc.114.134585 25794936 PMC4558658

[koaf108-B12] Cherry JM, Cartinhour SW, Goodman HM. AAtDB, anArabidopsis thaliana database. Plant Mol Biol Rep. 1992:10(4):308–309. 10.1007/BF02668902.

[koaf108-B13] Chopra R, Johnson EB, Daniels E, McGinn M, Dorn KM, Esfahanian M, Folstad N, Amundson K, Altendorf K, Betts K, et al Translational genomics using Arabidopsis as a model enables the characterization of pennycress genes through forward and reverse genetics. Plant J. 2018:96(6):1093–1105. 10.1111/tpj.14147 30394623

[koaf108-B14] Chopra R, Johnson EB, Emenecker R, Cahoon EB, Lyons J, Kliebenstein DJ, Daniels E, Dorn KM, Esfahanian M, Folstad N, et al Identification and stacking of crucial traits required for the domestication of pennycress. Nat Food. 2020:1(1):84–91. 10.1038/s43016-019-0007-z

[koaf108-B15] Chory J, Ecker JR, Briggs S, Caboche M, Coruzzi GM, Cook D, Dangl J, Grant S, Guerinot ML, Henikoff S, et al National Science Foundation-sponsored workshop report: “the 2010 project” functional genomics and the virtual plant. A blueprint for understanding how plants are built and how to improve them. Plant Physiol. 2000:123(2):423–426. 10.1104/pp.123.2.423 10859172 PMC1539254

[koaf108-B16] Chuang C-F, Meyerowitz EM. Specific and heritable genetic interference by double-stranded RNA in Arabidopsis thaliana. Proc Natl Acad Sci U S A. 2000:97(9):4985–4990. 10.1073/pnas.060034297 10781109 PMC18344

[koaf108-B17] Clough SJ, Bent AF. Floral dip: a simplified method for -mediated transformation of. Plant J. 1998:16(6):735–743. 10.1046/j.1365-313x.1998.00343.x 10069079

[koaf108-B18] Deal RB, Henikoff S. The INTACT method for cell type–specific gene expression and chromatin profiling in Arabidopsis thaliana. Nat Protoc. 2011:6(1):56–68. 10.1038/nprot.2010.175 21212783 PMC7219316

[koaf108-B19] Denyer T, Ma X, Klesen S, Scacchi E, Nieselt K, Timmermans MCP. Spatiotemporal developmental trajectories in the *Arabidopsis* root revealed using high-throughput single-cell RNA sequencing. Dev Cell. 2019:48(6):840–852.e5. 10.1016/j.devcel.2019.02.022 30913408

[koaf108-B20] Dombrecht B, Xue GP, Sprague SJ, Kirkegaard JA, Ross JJ, Reid JB, Fitt GP, Sewelam N, Schenk PM, Manners JM, et al MYC2 differentially modulates diverse jasmonate-dependent functions in Arabidopsis. Plant Cell. 2007:19(7):2225–2245. 10.1105/tpc.106.048017 17616737 PMC1955694

[koaf108-B21] Dutt M, Barthe G, Irey M, Grosser J. Transgenic citrus expressing an Arabidopsis NPR1 gene exhibit enhanced resistance against huanglongbing (hlb; citrus greening). PLoS One. 2015:10(9):e0137134. 10.1371/journal.pone.0137134 26398891 PMC4580634

[koaf108-B22] Feldmann KA, Marks MD, Christianson ML, Quatrano RS. A dwarf mutant of Arabidopsis generated by T-DNA insertion mutagenesis. Science. 1989:243(4896):1351–1354. 10.1126/science.243.4896.1351 17808268

[koaf108-B23] Fernández-Calvo P, Chini A, Fernández-Barbero G, Chico J-M, Gimenez-Ibanez S, Geerinck J, Eeckhout D, Schweizer F, Godoy M, Franco-Zorrilla JM, et al The Arabidopsis bHLH transcription factors MYC3 and MYC4 are targets of JAZ repressors and act additively with MYC2 in the activation of jasmonate responses. Plant Cell. 2011:23(2):701–715. 10.1105/tpc.110.080788 21335373 PMC3077776

[koaf108-B24] Fichman Y, Zandalinas SI, Peck S, Luan S, Mittler R. HPCA1 is required for systemic reactive oxygen species and calcium cell-to-cell signaling and plant acclimation to stress. Plant Cell. 2022:34(11):4453–4471. 10.1093/plcell/koac241 35929088 PMC9724777

[koaf108-B25] Fowler S, Thomashow MF. Arabidopsis transcriptome profiling indicates that multiple regulatory pathways are activated during cold acclimation in addition to the CBF cold response pathway. Plant Cell. 2002:14(8):1675–1690. 10.1105/tpc.003483 12172015 PMC151458

[koaf108-B26] Grant MR, Godiard L, Straube E, Ashfield T, Lewald J, Sattler A, Innes RW, Dangl JL. Structure of the Arabidopsis RPM1 gene enabling dual specificity disease resistance. Science. 1995:269(5225):843–846. 10.1126/science.7638602 7638602

[koaf108-B27] Grinstein JD . Salad days: pairwise gene edits food to topple nutrition barriers. GEN Biotechnol. 2023:2(1):5–9. 10.1089/genbio.2023.29078.jdg

[koaf108-B28] Halkier BA, Gershenzon J. Biology and biochemistry of glucosinolates. Annu Rev Plant Biol. 2006:57(1):303–333. 10.1146/annurev.arplant.57.032905.105228 16669764

[koaf108-B29] Hu Y, Jiang L, Wang F, Yu D. Jasmonate regulates the INDUCER OF CBF EXPRESSION–C-REPEAT BINDING FACTOR/DRE BINDING FACTOR1 cascade and freezing tolerance in Arabidopsis. Plant Cell. 2013:25(8):2907–2924. 10.1105/tpc.113.112631 23933884 PMC3784588

[koaf108-B30] Huala E, Dickerman AW, Garcia-Hernandez M, Weems D, Reiser L, LaFond F, Hanley D, Kiphart D, Zhuang M, Huang W, et al The Arabidopsis Information Resource (TAIR): a comprehensive database and web-based information retrieval, analysis, and visualization system for a model plant. Nucleic Acids Res. 2001:29(1):102–105. 10.1093/nar/29.1.102 11125061 PMC29827

[koaf108-B31] Jean-Baptiste K, McFaline-Figueroa JL, Alexandre CM, Dorrity MW, Saunders L, Bubb KL, Trapnell C, Fields S, Queitsch C, JT C. Dynamics of gene expression in single root cells of Arabidopsis thaliana. Plant Cell. 2019:31(5):993–1011. 10.1105/tpc.18.00785 30923229 PMC8516002

[koaf108-B32] Kim J-Y, Symeonidi E, Pang TY, Denyer T, Weidauer D, Bezrutczyk M, Miras M, Zöllner N, Hartwig T, Wudick MM, et al Distinct identities of leaf phloem cells revealed by single cell transcriptomics. Plant Cell. 2021:33(3):511–530. 10.1093/plcell/koaa060 33955487 PMC8136902

[koaf108-B33] Kranz AR, Kirchheim B. Genetic resources in Arabidopsis: computerized listing of seed bank material. Vol. 24. Frankfurt: Botanical Institute, J.W. Goethe-University, Arabidopsis Information Service; 1987. p. 1–167.

[koaf108-B34] Laibach F . *Arabidopsis thaliana* (L.) Heynh. als Objekt für genetische und entwicklungsphysiologische Untersuchungen. Bot Archiv. 1943:44:439–455.

[koaf108-B35] Lee KP, Liu K, Kim EY, Medina-Puche L, Dong H, Di M, Singh RM, Li M, Qi S, Meng Z, et al The m6A reader ECT1 drives mRNA sequestration to dampen salicylic acid–dependent stress responses in Arabidopsis. Plant Cell. 2024:36(3):746–763. 10.1093/plcell/koad300 38041863 PMC10896288

[koaf108-B36] Leutwiler LS, Hough-Evans BR, Meyerowitz EM. The DNA of Arabidopsis thaliana. Mol Gen Genet. 1984:194(1–2):15–23. 10.1007/BF00383491

[koaf108-B37] Levskaya A, Weiner OD, Lim WA, Voigt CA. Spatiotemporal control of cell signalling using a light-switchable protein interaction. Nature. 2009:461(7266):997–1001. 10.1038/nature08446 19749742 PMC2989900

[koaf108-B38] Li J-F, Norville JE, Aach J, McCormack M, Zhang D, Bush J, Church GM, Sheen J. Multiplex and homologous recombination–mediated genome editing in Arabidopsis and Nicotiana benthamiana using guide RNA and Cas9. Nat Biotechnol. 2013:31(8):688–691. 10.1038/nbt.2654 23929339 PMC4078740

[koaf108-B39] Liljegren S, Yanofsky M. Control of fruit dehiscence in Arabidopsis by indehiscent genes. United States Patent. Patent No. 6,998,517. 2006.

[koaf108-B40] Liljegren SJ, Roeder AHK, Kempin SA, Gremski K, Østergaard L, Guimil S, Reyes DK, MF Y. Control of fruit patterning in Arabidopsis by INDEHISCENT. Cell. 2004:116(6):843–853. 10.1016/S0092-8674(04)00217-X 15035986

[koaf108-B41] Liu J, Jung C, Xu J, Wang H, Deng S, Bernad L, Arenas-Huertero C, Chua N-H. Genome-wide analysis uncovers regulation of long intergenic noncoding RNAs in Arabidopsis. Plant Cell. 2012:24(11):4333–4345. 10.1105/tpc.112.102855 23136377 PMC3531837

[koaf108-B42] Lloyd AM, Barnason AR, Rogers SG, Byrne MC, Fraley RT, Horsch RB. Transformation of Arabidopsis thaliana with Agrobacterium tumefaciens. Science. 1986:234(4775):464–466. 10.1126/science.234.4775.464 17792019

[koaf108-B43] Long TA, Tsukagoshi H, Busch W, Lahner B, Salt DE, Benfey PN. The bHLH transcription factor POPEYE regulates response to iron deficiency in Arabidopsis roots. Plant Cell. 2010:22(7):2219–2236. 10.1105/tpc.110.074096 20675571 PMC2929094

[koaf108-B44] Marks MD, Feldmann KA. Trichome development in Arabidopsis thaliana. I. T-DNA tagging of the GLABROUS1 gene. Plant Cell. 1989:1(11):1043–1050. 10.2307/3869021 12359885 PMC159841

[koaf108-B45] Martin C, Butelli E. The purple tomato story; from laboratory bench to the consumer. ACS Food Sci Technol. 2025:5(1):19–28. 10.1021/acsfoodscitech.4c00692 39840404 PMC11744742

[koaf108-B46] McCallum CM, Comai L, Greene EA, Henikoff S. Targeted screening for induced mutations. Nat Biotechnol. 2000:18(4):455–457. 10.1038/74542 10748531

[koaf108-B47] McKelvie AD . Studies in the induction of mutations in *Arabidopsis thaliana* (L.) Heynh. Radiat Bot. 1963:3(2):105–123. 10.1016/S0033-7560(63)80032-0

[koaf108-B48] Mei S, Zhang M, Ye J, Du J, Jiang Y, Hu Y. Auxin contributes to jasmonate-mediated regulation of abscisic acid signaling during seed germination in Arabidopsis. Plant Cell. 2023:35(3):1110–1133. 10.1093/plcell/koac362 36516412 PMC10015168

[koaf108-B49] Meinke DW, Cherry JM, Dean C, Rounsley SD, Koornneef M. Arabidopsis thaliana: a model plant for genome analysis. Science. 1998:282(5389):662–682. 10.1126/science.282.5389.662 9784120

[koaf108-B50] Merkys AJ, Laurinavicius RS. Complete cycle of individual development in plants of Arabidopsis thaliana (L.) Heynh. aboard the salyut-7 orbital station. Dokl Bot Sci. 1983:271–273:68–70.

[koaf108-B51] Meyerowitz EM, Pruitt RE. Arabidopsis thaliana and plant molecular genetics. Science. 1985:229(4719):1214–1218. 10.1126/science.229.4719.1214 17770799

[koaf108-B52] Mindrinos M, Katagiri F, Yu G-L, Ausubel FM. The A. thaliana disease resistance gene RPS2 encodes a protein containing a nucleotide-binding site and leucine-rich repeats. Cell. 1994:78(6):1089–1099. 10.1016/0092-8674(94)90282-8 7923358

[koaf108-B53] Montes C, Zhang J, Nolan TM, Walley JW. Single-cell proteomics differentiates Arabidopsis root cell types. New Phytol. 2024:244(5):1750–1759. 10.1111/nph.19923 38923440

[koaf108-B54] Nishimura K, Fukagawa T, Takisawa H, Kakimoto T, Kanemaki M. An auxin-based degron system for the rapid depletion of proteins in nonplant cells. Nat Methods. 2009:6(12):917–922. 10.1038/nmeth.1401 19915560

[koaf108-B55] Nobori T, Oliva M, Lister R, Ecker JR. Multiplexed single-cell 3D spatial gene expression analysis in plant tissue using PHYTOMap. Nat Plants. 2023:9(7):1026–1033. 10.1038/s41477-023-01439-4 37308583 PMC10356616

[koaf108-B56] Okushima Y, Overvoorde PJ, Arima K, Alonso JM, Chan A, Chang C, Ecker JR, Hughes B, Lui A, Nguyen D, et al Functional genomic analysis of the AUXIN RESPONSE FACTOR gene family members in Arabidopsis thaliana: unique and overlapping functions of ARF7 and ARF19. Plant Cell. 2005:17(2):444–463. 10.1105/tpc.104.028316 15659631 PMC548818

[koaf108-B57] O’Malley RC, Huang SC, Song L, Lewsey MG, Bartlett A, Nery JR, Galli M, Gallavotti A, Ecker JR. Cistrome and epicistrome features shape the regulatory DNA landscape. Cell. 2016:165(5):1280–1292. 10.1016/j.cell.2016.04.038 27203113 PMC4907330

[koaf108-B58] Phippen W, Rhykerd R, Sedbrook J, Cristine H, Csonka S. From farm to flight: covercress as a low carbon intensity cash cover crop for sustainable aviation fuel production. a review of progress towards commercialization. Frontiers (Boulder). 2022:10:793776. 10.3389/fenrg.2022.793776

[koaf108-B59] Rédei GP . Arabidopsis as a genetic tool. Annu Rev Genet. 1975:9(1):111–127. 10.1146/annurev.ge.09.120175.000551 1108762

[koaf108-B60] Reinholz E . Röntgenmutationen bei Arabidopsis Thaliana (L) heynh. Naturwissenschaften. 1947:34(1):26–28. 10.1007/BF00633319

[koaf108-B61] Röbbelen G . Wirkungsvergleich zwischen Äthylmethansulfonat und Röntgenstrahlen im Mutationsversuch mit*Arabidopsis thaliana*. Naturwissenschaften. 1962:49(3):65. 10.1007/BF00595396

[koaf108-B62] Röbbelen G . The laibach standard collection of natural races. Arabidopsis Information Service. 1965:2:36–42.

[koaf108-B63] Ryu KH, Huang L, Kang HM, Schiefelbein J. Single-cell RNA sequencing resolves molecular relationships among individual plant cells. Plant Physiol. 2019:179(4):1444–1456. 10.1104/pp.18.01482 30718350 PMC6446759

[koaf108-B64] Schena M, Shalon D, Davis RW, Brown PO. Quantitative monitoring of gene expression patterns with a complementary DNA microarray. Science. 1995:270(5235):467–470. 10.1126/science.270.5235.467 7569999

[koaf108-B65] Schwab R, Ossowski S, Riester M, Warthmann N, Weigel D. Highly specific gene silencing by artificial microRNAs in Arabidopsis. Plant Cell. 2006:18(5):1121–1133. 10.1105/tpc.105.039834 16531494 PMC1456875

[koaf108-B66] Shen J, Zhang J, Zhou M, Zhou H, Cui B, Gotor C, Romero LC, Fu L, Yang J, Foyer CH, et al Persulfidation-based modification of cysteine desulfhydrase and the NADPH oxidase RBOHD controls guard cell abscisic acid signaling. Plant Cell. 2020:32(4):1000–1017. 10.1105/tpc.19.00826 32024687 PMC7145499

[koaf108-B67] Shulse CN, Cole BJ, Ciobanu D, Lin J, Yoshinaga Y, Gouran M, Turco GM, Zhu Y, Malley O, Brady RC, et al High-throughput single-cell transcriptome profiling of plant cell types. Cell Rep. 2019:27(7):2241–2247.e4. 10.1016/j.celrep.2019.04.054 31091459 PMC6758921

[koaf108-B68] Silva KJP, Mahna N, Mou Z, Folta KM. NPR1 as a transgenic crop protection strategy in horticultural species. Hortic Res. 2018:5(1):15. 10.1038/s41438-018-0026-1 29581883 PMC5862871

[koaf108-B69] Simmons CR, Lafitte HR, Reimann KS, Brugière N, Roesler K, Albertsen MC, Greene TW, Habben JE. Successes and insights of an industry biotech program to enhance maize agronomic traits. Plant Sci. 2021:307:110899. 10.1016/j.plantsci.2021.110899 33902858

[koaf108-B70] Stief A, Altmann S, Hoffmann K, Pant BD, Scheible W-R, Bäurle I. Arabidopsis miR156 regulates tolerance to recurring environmental stress through SPL transcription factors. Plant Cell. 2014:26(4):1792–1807. 10.1105/tpc.114.123851 24769482 PMC4036586

[koaf108-B71] Sunkar R, Zhu J-K. Novel and stress-regulated microRNAs and other small RNAs from Arabidopsis. Plant Cell. 2004:16(8):2001–2019. 10.1105/tpc.104.022830 15258262 PMC519194

[koaf108-B72] Thalmann M, Pazmino D, Seung D, Horrer D, Nigro A, Meier T, Kölling K, Pfeifhofer HW, Zeeman SC, Santelia D. Regulation of leaf starch degradation by abscisic acid is important for osmotic stress tolerance in plants. Plant Cell. 2016:28(8):1860–1878. 10.1105/tpc.16.00143 27436713 PMC5006701

[koaf108-B73] The Arabidopsis Genome Initiative . Analysis of the genome sequence of the flowering plant Arabidopsis thaliana. Nature. 2000:408(6814):796–815. 10.1038/35048692 11130711

[koaf108-B74] Titova NN . Poiski rastitel'noy drosofily. (search of plant *Drosophila*). Sovetsk Bot. 1935:2:61–67.

[koaf108-B75] USDA APHIS . Citrus x sinensis transgenic lines tolerant to citrus greening disease. 2023 [accessed 2025 March 28]. https://www.aphis.usda.gov/sites/default/files/23-213-01rsr.pdf.

[koaf108-B76] Wei L-H, Song P, Wang Y, Lu Z, Tang Q, Yu Q, Xiao Y, Zhang X, Duan H-C, Jia G. The m6A reader ECT2 controls trichome morphology by affecting mRNA stability in Arabidopsis. Plant Cell. 2018:30(5):968–985. 10.1105/tpc.17.00934 29716990 PMC6002187

[koaf108-B77] Winter D, Vinegar B, Nahal H, Ammar R, Wilson GV, Provart NJ. An “electronic fluorescent pictograph” browser for exploring and analyzing large-scale biological data sets. PLoS One. 2007:2(8):e718. 10.1371/journal.pone.0000718 17684564 PMC1934936

[koaf108-B78] Yano M, Katayose Y, Ashikari M, Yamanouchi U, Monna L, Fuse T, Baba T, Yamamoto K, Umehara Y, Nagamura Y, et al Hd1, a major photoperiod sensitivity quantitative trait locus in rice, is closely related to the Arabidopsis flowering time gene CONSTANS. Plant Cell. 2000:12(12):2473–2483. 10.1105/tpc.12.12.2473 11148291 PMC102231

[koaf108-B79] Yaschenko AE, Alonso JM, Stepanova AN. Arabidopsis as a model for translational research. Plant Cell. 2024:37(5):koae065. 10.1093/plcell/koae065 PMC12082644 38411602

[koaf108-B80] Zanetti ME, Chang I-F, Gong F, Galbraith DW, Bailey-Serres J. Immunopurification of polyribosomal complexes of Arabidopsis for global analysis of gene expression. Plant Physiol. 2005:138(2):624–635. 10.1104/pp.105.059477 15955926 PMC1150383

[koaf108-B81] Zhang T-Q, Xu Z-G, Shang G-D, Wang J-W. A single-cell RNA sequencing profiles the developmental landscape of *Arabidopsis* root. Mol Plant. 2019:12(5):648–660. 10.1016/j.molp.2019.04.004 31004836

[koaf108-B82] Zhang Y, Butelli E, Alseekh S, Tohge T, Rallapalli G, Luo J, Kawar PG, Hill L, Santino A, Fernie AR, et al Multi-level engineering facilitates the production of phenylpropanoid compounds in tomato. Nat Commun. 2015:6(1):8635. 10.1038/ncomms9635 26497596 PMC4639801

[koaf108-B83] Zhong R, Lee C, Zhou J, McCarthy RL, Ye Z-H. A battery of transcription factors involved in the regulation of secondary cell wall biosynthesis in Arabidopsis. Plant Cell. 2008:20(10):2763–2782. 10.1105/tpc.108.061325 18952777 PMC2590737

[koaf108-B84] Zhou J, Lee C, Zhong R, Ye Z-H. MYB58 and MYB63 are transcriptional activators of the lignin biosynthetic pathway during secondary cell wall formation in Arabidopsis. Plant Cell. 2009:21(1):248–266. 10.1105/tpc.108.063321 19122102 PMC2648072

