# Supplementary figures and images for: A timeline of discovery and innovation in Arabidopsis
Source: Plant Cell. 2025 May 5;37(5):koaf108. doi: 10.1093/plcell/koaf108 (PMC12123313; doi:10.1093/plcell/koaf108)

# A Timeline of Discovery and Innovation in Arabidopsis

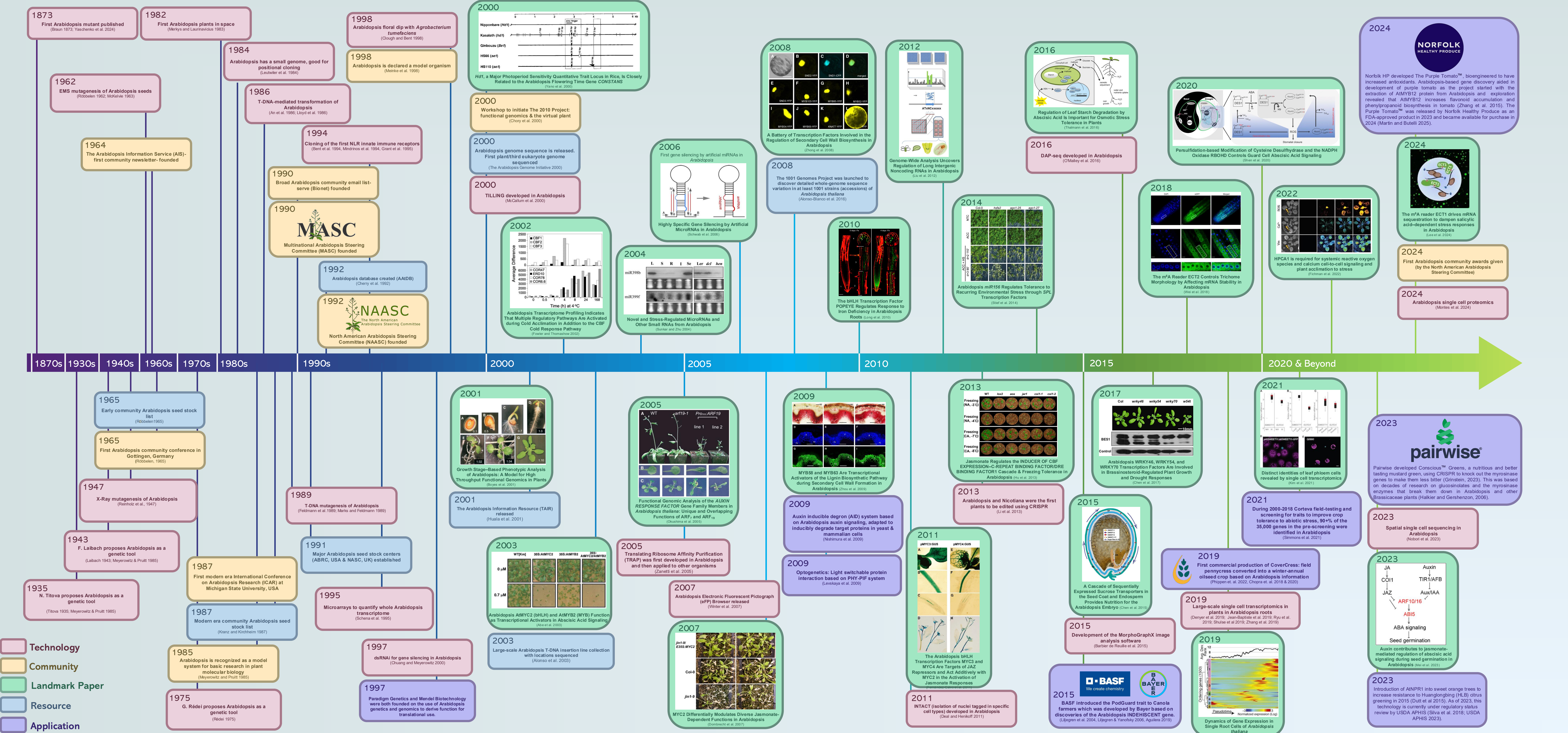

Supplement: koaf108_Supplementary_Data [file koaf108_supplementary_data.pdf]
